# Scientific Items

**Published:** 1881-03-20

**Authors:** 


### SCIENTIFIC ITEMS.

**Luminous Paint.**—We have already given some account of Balmain's luminous paint, patented not long ago in England. We do not know whether any experiments have been made with it in this country, but it is being tested in various ways in England, and thus far with very satisfactory results. It has been applied to ceilings as a water-wash, and the effect by night is that of "a subdued light, every object in the room being clearly visible." The luminosity, as our readers will remember, is excited by ordinary daylight, and its effects continue about thirteen hours, so that it is well adapted for bed-rooms, passages that are dark at night, and other places where the use of lamps would be objectionable or inconvenient. The London *Building News* says:

For staircases and passages a mere band of the paint will serve as a guide, and costs but a trifle. For out-door purposes the oil paint is used, but for ceilings and walls the luminous paint mixed with water and special size can be used, the same as ordinary whitewash, and presents a similar appearance in the daylight. We have previously given a short account of the many applications of which this paint is capable, but the comparatlvely recent discovery that it can be applied as ordinary whitewash, considerably expands the field of its usefulness. Sheets of glass coated with the paint form Aladdin's lamps, which are in use in some of the vessels of the navy, at the Walham Powder Factory, at Young's Paraffin works, and in the spirit vaults of several docks; but now that, by increased production and the use of water as the medium, the cost is reduced by one-half, it will probably be extensively used for painting walls and ceilings. The ordinary form of oil paint has already been applied in many ways to statues and busts, to toys, to clock-faces, to name-plates and numbers on house-doors, and to notice-boards, such as "mind the steps," "to let," etc. The paint emits light without combustion, and therefore does not vitiate the atmosphere. Several experimental carriages are now running on different railways, the paint being used instead of lamps, which are necessary all day on account of the line passing through occasional tunnels. —*Boston Journal of Chemistry.*

**A "National Radiant" Fluid.**—During the past month a "trial" was given in this city of an alleged substitute for kerosene oil, which is represented to cost much less, and to be perfectly "non-explosive." The usual empirical experiments to prove the safety of this mixiure were made, as lighting it with matches, dropping burning coals into it, etc. It is stated that the base of this new fluid is naptha, with the addition of thirteen cheap ingredients. Among these are potatoes, salt, alum, sulphuric acid, and other substances, all of which might be replaced with soot or sand, so far as they prevent explosions. It may sometime become generally known that there is no possible substitute for good standard kerosene, and that naptha is still dangerous, no matter how many vegetables are stewed up with it.

This dangerous mixture is manufactured in Cleveland, Ohio, and it is said that a large quantity is to be put upon the market, Our readers will take warning, and give the new "National Radiant" oil as wide a berth as if it were so much nitro-glycerine. The only safe oil, as has been intimated, is standard kerosene; and it is in the end the cheapest, as doctors' and undertakers' bills foot up much more than any saving made in purchasing cheap naptha under the name of "Radiant Fluid."—*Ibid.*

**Toughened Lamp Chimneys.**—The following recipe is from a Leipzig journal devoted to the glass interest: Place your tumblers, chimneys, or vessels which you desire to keep from cracking in a pot filled with cold water, add a little cooking salt, allow the mixture to boil well over a fire, and then cool slowly. Glass treated in this way is said not to crack even if exposed to very sudden changes of temperature. Chimneys are said to become very durable by this process, which may also be extended to crockery, stoneware, porcelain, etc. The process is simply one of annealing, and the slower the process, especially the cooling portion of it, the more effective will be the work.—*Ibid.*

**Preserving Wood.**—The improved French method of preserving wood by the application of lime is said to work well. The plan is to place the planks in a pile in the tank and to put over all a layer of quicklime, which is gradually slacked with water. Timber for mines requires about a week to be thoroughly impregnated, and other wood more or less time, according to its thickness. The material acquires remarkable consistence and hardness, it is stated, on being subjected to this simple process, and the assertion is made that it will never rot. Beechwood prepared in this way for hammers and other tools for iron-work is found to acquire the hardness of oak, without parting with any of its well-known elasticity or toughness, and it also lasts longer. —*Ibid.*

**Providence of Bees.**—The Melbourne correspondent of the Dundee *Advertiser* narrates the following interesting proof of the provident and far-seeing instinct of bees: Turning from men to insects, a singular circumstance is reported from a hot, dry valley in New South Wales. Last year the drought there was of long duration, and the denizens of the apiaries suffered much from it. This year the bees have made provision against a similar emergency. They have filled a large number of the external cells in every hive with pure water instead of honey. It is thought that the instinct of the little creatures leads them to anticipate a hot summer. But that they should have gone further, and, by an act which, as far as I know, is without precedent in the habits and customs of their tribes, have created reservoirs to tide over the water famine, is a noteworthy fact indeed.—*Ex.*

**A New Oxide of Boron.**—From recent experiment and analyses in France, it would appear that there exists a combination of boron with oxygen which has hitherto escaped the notice of chemists. The new boric acid is denoted by $B_2O_4$, and being the most oxygenated compound of boron known, has been named *perboric acid.*—*Ex.*

